# Spermatorrhœa

**Published:** 1879-07

**Authors:** 


### Books Reviewed.

*Spermatorrhœa.* By ROBERTS BARTHOLOW, M. D. Fourth Edition, Revised. Wm. Wood & Co.

The subject of spermatorrhœa is presented by Prof. Roberts Bartholow in an attractive monograph. The author is refined in his vocabulary, clear in his diction, and the paper embodies the more advanced views of the profession upon this topic, which physicians are coming to appreciate, to the disadvantage of the "Merry Andrew."

Lallemand has been the principal source of information on Spermatorrhœa for the past forty years, and, in this production the author seems justified in believing that he has supplied a want long felt.

His views of the pathology of the disorder are radically opposed to Lellemand's, and something the same may be said of his treatment. Bartholow considers the malady a neurosis, and upon this basis establishes his therapeusis. The analysis of the subject is complete and symmetrical, dividing it into true spermatorrhœa, spermatorrhœa as a symptom of some centric nervous lesion, and false or immaginary spermatorrhœa.

To the causes of true spermatorrhœa, the author devotes twenty-four pages which bear evidence of thoughtful investigation, being the most philosophical *expose* of the causes that we remember of seeing, and offering some rather original views which are supported by numerous and varied citations that are neither discursive nor prolix, but to the point, and so selected that they afford the writer a strong position and M. Lallemand's school a feeble one.

While submitting that the disorder is practically always a neurosis, in contradiction to Lallemand, who maintains that its pathology consists in a produced irritation or inflammation of the prostatic portion of the urethra and the seminal ducts, he agrees with the French author that the malady itself is usually the sequence of sexual excesses—in most cases from masturbation. But it is contended that inordinate sexual indulgence cannot have the effect to produce prostatitis (and allied conditions,) unless gonorrhœa be already existing, and refers to the opinion of M. Raige, Delorme, and Mr. Thompson for authority; that Lallemand's theory which has crept into so many text books, is not only untenable, as the authors dissections prove, but conducive to improper treatment also; and that structural alterations are not causative, only as they increase disorders of the nervous apparatus.

Medication is mostly confined to tonic and anaphrodisiac measures—and Bromide of Potassium is the favorite remedy.

Some salutary sanitary rules are given that supersede all else in the treatment of true spermatorrhœa in our estimation.

After a few lines on the treatment of impotence, the Professor closes his deservedly successful little book with a number of formulæ, the most hackneyed of which, it would appear, are the most valuable.

